# Secular trends and determinants of *ad libitum* energy intake measured in a research setting from 1999-2020

**DOI:** 10.3389/fnut.2024.1374386

**Published:** 2024-06-12

**Authors:** Mary M. Ahern, Emma J. Stinson, Paolo Piaggi, Jonathan Krakoff, Susanne B. Votruba

**Affiliations:** Obesity and Diabetes Clinical Research Section, Phoenix Epidemiology and Clinical Research Branch, National Institute of Diabetes and Digestive and Kidney Diseases, National Institutes of Health, Phoenix, AZ, United States

**Keywords:** dietary intake, secular trends, seasonality, spontaneous physical activity, self-report dietary intake

## Abstract

**Introduction:**

Historically, secular and seasonal trend analyses have been examined using self-report measures of intake. Rarely are objective measures and known determinants of dietary intake used in these analyses. Our objective was to quantify the seasonal and secular differences in an objective *ad libitum* intake paradigm while considering the contribution of determinants, such as fat-free mass (FFM) index and spontaneous physical activity (SPA) limited to the restricted space of a whole-room calorimeter.

**Methods:**

For this study, recruitment of *N* = 292 healthy, diabetes free, adults occurred from 1999 to 2020. Assessment during their 10-day stay included body composition (by DXA), SPA (by an approximately 24-h stay in whole-room calorimetry), and *ad libitum* intake (by a vending machine for 3 days). This secondary analysis used general linear models (GLM) to investigate secular and seasonal differences while adjusting for sex, age, FFM index, FM (fat mass) index, SPA, and race/ethnicity.

**Results:**

FFM index and SPA were positively associated with all intake measures (*p* < 0.05). In all adjusted seasonal models, season did not affect intake. Adjusted secular trends models (kcals/year) demonstrated a decrease in total kcals (*β* = −55), intake as percent weight maintaining energy needs (*β* = −2), protein kcals (*β* = −10), fat kcals (*β* = −27), and carbohydrates kcals (*β* = −22) (all *p* < 0.05). After further adjustment for SPA, significance remained in all intake measures (*p* < 0.05). Secular trends in body composition revealed no changes in weight, BMI, and percent body fat (all *p* > 0.20).

**Discussion:**

Our results indicate that over time, *ad libitum* intake decreased in this controlled research setting and remained significant even after accounting for positive determinants of intake. A significant *ad libitum* decrease, coupled with no change in body composition, may highlight a participant bias toward calorie restriction in a controlled setting over time and deserves further investigation.

## 1 Introduction

Changes in food intake patterns may vary over different temporal scales, including seasonally and over more extended periods (secular trends), and are influenced by shifts in agricultural, cultural, socioeconomic, and environmental factors (1–3). Understanding the temporal changes in energy intake may help elucidate mechanistic or physiological reasons for the rise in obesity rates over the last few decades (4). Rates of obesity have risen around the world since the 1980s, with the current prevalence in American adults at approximately 42% (4). Examining changes in components of energy balance principles may help understand this increase. Energy balance considers the relationship between two components of human metabolism: energy intake and expenditure (5). Fundamentally, the energy balance equation argues that energy intake in excess of energy expenditure leads to weight gain (5). Therefore, understanding the role of energy intake in secular and seasonal models will be important to understanding how trends in dietary intake have changed over time.

Evidence for a seasonal effect on dietary intake is inconsistent (6, 7). A meta-analysis showed slightly higher intake in winter compared to other seasons (7). Another study in the US also demonstrated increases in overall dietary intake in winter and attributed this to a holiday season effect (8). However, results are not consistent across populations. Yoshimura et al. (6) demonstrated that participants in Japan ate significantly fewer overall calories, less protein, and less fat in winter than in autumn. Still, other studies have found no effect of seasonality on intake. Bernstein et al. (9) found no relationship between seasons and the intake of macronutrients, micronutrients, or food groups. Ma et al. (10) demonstrated slight fluctuations in total energy intake, fat, and carbohydrate intake throughout the year but no clear seasonal pattern.

Research supporting changes in dietary intake over time (secular trends) is mixed as well. In the last few decades, the impact of diet on obesity has been a huge concern of governments and public health officials. In the United States alone, we have implemented several nutrition policies to try to impact dietary intake over the years, including improving school lunches, taxing soda, and adding calories to menu items at restaurants, all in an effort to improve nutrition and decrease unhealthy calorie intake (11). Despite all these efforts, individuals still eat more today than they were decades ago. Le et al. (12), using food availability data published by the Food and Agriculture Organization at the United Nations, demonstrated an increase in the global average calories consumed per person from approximately 2,250 in 1960 to 2,800 in the 2010s. Therefore, in order to understand trends in dietary intake, it is important to look at secular trends in addition to seasonal ones.

The National Health and Nutrition Examination Survey (NHANES) data from 1999 to 2016 reported a decrease in the percent energy intake of carbohydrates and an increase in the percent intake of protein and fat (13). During this time, NHANES data also recorded increased high-quality carbohydrates, saturated and unsaturated fatty acids, and an overall rise in the Healthy Eating Index score (13). Despite an apparent increase in diet quality, intakes of low-quality carbohydrates and saturated fats remained high (13). Using NHANES data from 1971 to 2008, Brown et al. (1) reported decreased total fat and protein intake and increased carbohydrates, overall intake, leisure time, physical activity, and BMI. This inconsistency in secular trends results may be due to the inherent limitations and bias of self-reported dietary intake measures, such as recall bias and measurement error (14). Historically, secular and season trend analyses, such as with NHANES collected data, have used subjective self-reported measures of dietary intake (14).

Additionally, our group has previously identified temporal decreases in spontaneous physical activity (SPA) and a positive association between SPA, as used in the constrained space of the whole-room calorimeter, and energy intake (5, 15). Briefly, in our study, SPA is a measure of physical activity that is associated with fidgeting-like behaviors and small preferences for movement, such as standing instead of sitting (15). Even though it is a small component of overall energy expenditure, it is highly variable and easily measured in certain study paradigms (like a metabolic chamber) (15). Thus, SPA was included in models to investigate how temporal factors (i.e., seasonality and secular trends over time) affect the relationship between SPA and food intake. Lastly, it has been shown that fat-free mass (FFM) index is a significant positive predictor of energy intake and should also be included in the models (16).

The primary aim of the current study was to assess the effects of seasonality and secular trends on *ad libitum* dietary intake as objectively quantified in an inpatient setting. The secondary aim was to evaluate whether the relationships between known determinants of energy intake, SPA, and fat-free mass (FFM) index account for these trends (5, 16). We hypothesize that there will be a time-related change in dietary intake on our vending machine paradigm, which will be impacted by the inclusion of a measurement of physical activity (SPA) and body composition (FFM index).

## 2 Materials and methods

### 2.1 Recruitment

Recruitment for this study occurred at the NIDDK Obesity and Diabetes Clinical Research section in Phoenix, Arizona. Participants were recruited by NIDDK staff through flyers, newspaper ads, the internet (clinicaltrials.gov), and word of mouth. It was part of a larger inpatient study, the Food Intake Phenotype Study, that assessed changes in food preferences and intake, which has been actively recruiting since 1999 (ClinicalTrails.gov identifier NCT00342732) (17). All participants consented to participate in this study by giving written informed consent. This was approved by the NIH IRB.

### 2.2 Inclusion and exclusion criteria

**FIGURE 1:**
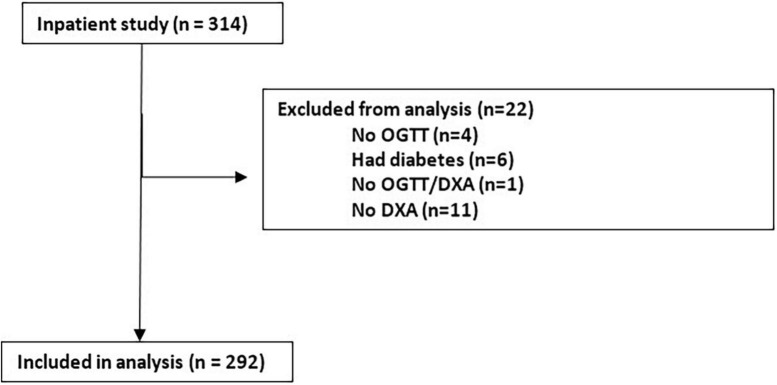
Inclusion and exclusion criteria for participants included in this secular and seasonal analysis. Based on valid vending machine information, *N* = 314 participants in Phoenix, Arizona, were selected for this analysis. *N* = 22 were excluded from the analysis for the reasons listed above. Leaving *N* = 292 participants for analysis.

Participants selected for analysis from 1999 to 2020 were healthy adults (based on falling within the normal range for screening labs, medical history, and a physical exam), free from diabetes (based on oral glucose tolerance test (OGTT) on day 4). *N* = 314 individuals were available for this analysis based on available and complete vending machine information (Figure 1). Participants were further excluded from this analysis if OGTT results revealed they had diabetes (*n* = 6) or missing data for the OGTT (*n* = 5). Lastly, participants (*n* = 11) were excluded for missing DXA information such as height and weight. In total, *n* = 22 of the initial 314 were excluded for the above reasons, leaving *n* = 292 for subsequent analysis. All analyses with SPA were conducted on a subset, *n* = 206, as some participants were missing SPA data (*n* = 86). No metabolic chamber dates were recorded from November 2005 to September 2009; however, the recruitment and data collections methods did not change over this time.

### 2.3 Data collection

Once admitted to the inpatient study floor at the NIH Phoenix, patients began a weight-maintaining diet for the first three days of their ten-day inpatient stay. The weight-maintaining energy needs (WMEN; 50% carbohydrates, 30% fat, and 20% protein) were first calculated using Ferraro et al.’s equation based on weight and gender and then adjusted daily by the research dietitian to ensure stable weight throughout the baseline period before volunteers had access to the vending machines (18, 19). During this time, a DXA (DPX-1, Lunar Radiation Corp, Madison, Wisconsin) machine assessed body composition measurements such as fat-free mass (FFM) and fat mass (FM). These measurements were used to determine the FFM index and FM index, which considers height in addition to overall fat-free mass or fat mass (20). Due to the long recruitment period of these studies, from 1999 to 2020, different DXA machines were used. Therefore, values were standardized across DXA machines using comparative equations (21, 22). Next, participants spent roughly twenty-four hours in a metabolic calorimeter, which measured twenty-four-hour energy expenditure and its components, including SPA (23). While not a measure of overall physical activity, SPA reflects small restless movements participants make in the respiratory chambers, like sitting to standing. During their stay in the metabolic calorimeter, participants were fed a weight-maintaining diet and asked not to exercise.

### 2.4 Dietary intake collection

Following the stay in the metabolic calorimeter, participants ate *ad libitum* using an automated vending machine paradigm for three days, as previously described (17). Each participant was assigned their own vending machine, and the foods selected for that vending machine were individualized for each participant based on their answers to a hedonic food assessment (24). Foods that appeared on this assessment ranged from single food items such as eggs or spinach to full meals like spaghetti and meat sauce. A full list of food items can be seen in Table 1. Barring some changes in the availability of certain food items, the food list has been consistent since the beginning of the study. Participants then selected these foods by completing an 80-item Food Selection Questionnaire containing ordinary mealtime foods by rating their preference for the items listed on a scale from 1 (extremely dislike) to 9 (extremely like), with 5 being neutral. Foods selected for the vending machine ranged from 4 to 8 to serve participants food they liked while trying not to encourage overconsumption (24). Before being placed in the vending machines in the morning, all selected food and packaging for the day were inventoried and weighed.

**TABLE 1:** List of 77 food items offered in the vending machine protocol.

Food item		
Pizza	Oatmeal	Ham
Cheeseburger	Chocolate pudding	Green beans
Orange	English muffin	Potato salad
Fried chicken	Corn flakes and milk	Chicken with pasta
Eggs	Refried beans	Popcorn
Baked potato	Granola bar	Apple pie
Corn	Cup of noodles	Sliced turkey
Spaghetti with sauce	Bagel	Blueberry muffins
Chicken pot pie	Rice Krispies and milk	KitKat bar
Barbecue wings	Pinto beans	Nestle crunch
French toast	Peanuts	Cheez-Its
Beef stew	Sausage McMuffin	Tortilla chips
Sausage	Ritz crackers	Chicken noodle soup
Reese’s cups	Rice Krispies treats	Crackers
Cheddar cheese	Cinnamon bun	Pretzels
Chili with beans	Cottage cheese	Baby Ruth
Pancakes	Chocolate donut	Spinach
Apples	Jello	Pork and beans
Chicken nuggets	Apple sauce	Bologna
Stuffed baked potato	Croissant	Raisins
Potato chips	Yogurt	Fig Newtons
Peanut M and M’s	Corned beef hash	Gummy bears
Cooked rice	Cheesecake	Fruit roll-ups
Tater tots	Macaroni salad	Mushroom soup
Peaches	Canned tuna	Sugar wafers
Doritos	Fudge cookies	

During the three days in which patients were encouraged to eat *ad libitum* from the vending machines, study participants were required to eat alone in a specified vending machine room without distractions such as TV, personal devices, or phones. Typical condiments and bread were available in this room. When they were hungry, participants chose the food they wished to eat, and time was recorded. All packaging and any leftover food were reweighed after the meal to calculate an accurate intake for each eating episode (including bread and condiment intake). The reproducibility of this paradigm has been validated in previous studies, with an ICC of 0.90 (17, 19).

Macronutrient intake assessment was completed using CBORD Professional Diet Analyzer Program (CBORD Inc., Ithaca, NY, USA) and Food Processor (version 10.0.0; ESHA Research, Salem, OR, USA) (25, 26). Intake variables on the vending machines, such as total energy intake, carbohydrate kcal intake, protein kcal intake, and fat kcal intake, were averaged for the three days inpatient and are reported as the mean calorie intake, which was also expressed as a percentage of WMEN (%WMEN).

### 2.5 Categorizing seasonality

Seasonality was defined using temperatures from the Global Daily Climatology Network dataset archived with the US NOAA/National Climatic Data Center. See Aydin et al. (27) for a more detailed explanation of how the seasonal cut-offs were determined. Here, season definitions are winter defined as December 21st to March 20th, spring as March 21st to June 20th, summer as June 21st to September 20th, and fall as September 21st to December 20th (27).

### 2.6 Demographic data collection

Race/ethnicity, sex, and age were collected via self-report on a demographic questionnaire during intake. Race/ethnicity was divided into four distinct categories: Indigenous Americans, White, Black, and Other (the other category comprised Asian, Hispanic, and people who identified as multiple races). Indigenous Americans accounted for a large percentage of participants. Therefore, sensitivity analyses were also conducted for all models, including only Indigenous Americans, which did not change the results (data not shown).

### 2.7 Statistical analysis

SAS (version 9.4, SAS Institute Inc., Cary, NC, USA) was used for the statistical analysis (28). An alpha of 0.05 was set as the significance level for all tests. Normally distributed data are expressed in mean +/− standard deviation (SD). First, dietary intake measures were assessed without seasonal or secular trends variables to understand the impact of the determinants of dietary intake in the models. This was done using general linear models (GLMs) adjusted for sex, age, race/ethnicity, FFM index, FM index, and SPA in all intake measurements. Next, separate GLMs were used to assess seasonality and secular trends in food intake by including either season (seasonality: winter = reference) or date (secular trends) in the abovementioned model. Models were further adjusted for SPA to assess its relationship with time-related trends and dietary intake. Separate general linear models assessed secular trends in body composition measures (weight, percent body fat, and BMI) over time while adjusting for sex, age, and race/ethnicity. R-squared for certain GLMs were reported in the results as well. All secular trends data was reported in change by year. Results were also quantified using partial correlations (partial *r*) adjusting for the same covariates. Beta coefficients for these models are abbreviated with “*β*” representing a change of kcal or percent for a 1 unit change in the predictors.

Our research unit has two respiratory chambers. To ensure this did not impact the data, a sensitivity analysis was run controlling for the chamber, and the results remained the same (data not shown). Twenty-four-hour energy expenditure, another major determinant of energy intake, was also adjusted for all models in place of body composition measurements, and results remained the same (data not shown).

## 3 Results

The following analysis included a total of 292 participants (Table 2). The majority were male (*n* = 178, 61%), Indigenous American (*n* = 171, 59%), with obesity (*n* = 145, 50%) and a mean BMI of 31.6 ± 8.03. Participants were roughly evenly distributed across seasons, with fall having the most participants (*n* = 79), followed by spring (*n* = 76), winter (*n* = 75), and summer (*n* = 62). The average total energy intake of all the participants on the vending machines was 3,896 ± 1,375 kcal/day, and the average percent of weight-maintaining diet eaten of 140% ± 46%, demonstrating the documented propensity of participants to overeat on this reproducible vending machine paradigm (17).

**TABLE 2:** Demographics, anthropometrics, and intake of 292 healthy study participants by season collected from 1999 to 2020.

Demographics	Fall	Winter	Spring	Summer	Total
*n* (%)	79 (27%)	75 (26%)	76 (26%)	62 (21%)	292
Age (years)	34.5 (11.1)	36.3 (10.5)	34.9 (10.1)	38.2 (10.5)	35.9 (10.6)
**Race/ethnicity, *n* (%)**					
AI/AN	51 (64.6%)	46 (61.3%)	38 (50%)	36 (58.1%)	171 (58.6%)
White	20 (25.3%)	19 (25.3%)	31 (40.8%)	14 (22.6%)	84 (28.8%)
AA	2 (2.5%)	6 (8%)	3 (3.9%)	5 (8.1%)	16 (5.5%)
Other	6 (7.6%)	4 (5.3%)	4 (5.3%)	7 (11.3%)	21 (7.2%)
**Sex**					
Male	44 (55.7%)	47 (62.7%)	47 (61.8%)	40 (64.5%)	178 (61%)
Female	35 (44.3%)	28 (37.3%)	29 (38.2%)	22 (35.5%)	114 (39%)
**Body composition measurements**					
FFM (kg)	61.2 (14.5)	60.9 (14.4)	62.3 (10.4)	59.8 (14.2)	61.1 (13.4)
FM (kg)	32.3 (15.4)	27.9 (12.4)	31.0 (14.3)	25.6 (12.8)	29.4 (14.0)*
FFM index (kg/m)^2^	21.9 (4.1)	20.8 (3.8)	21.4 (3.1)	20.5 (3.5)	21.2 (3.7)
FM index (kg/m)^2^	11.8 (5.9)	9.8 (4.7)	10.9 (5.5)	8.9 (4.2)	10.4 (5.2)*
BMI (kg/m^2^)	33.7 (9.1)	30.5 (7.5)	32.4 (7.8)	29.4 (6.8)	31.6 (8.0)*
Height (cm)	166.6 (9.0)	170.6 (9.6)	170.4 (8.8)	169.9 (9.0)	169.3 (9.2)*
Body weight (kg)	93.5 (26.2)	88.8 (23.0)	93.3 (20.8)	85.4 (23.6)	90.5 (23.6)
Body fat (%)	33.4 (9.0)	30.6 (8.9)	31.9 (9.3)	29.0 (8.5)	31.4 (9.0)*
**Dietary intake variables**					
Total intake (kcal)	3,802 (1,556)	4,051 (1,402)	4,053 (1,276)	3,635 (1,181)	3,896 (1,375)
Total intake (% WMEN)	135.0 (50.2)	146.1 (45.8)	145.6 (44.3)	131.9 (39.7)	139.9 (45.6)
WMEN (kcal)	2,795 (283)	2,752 (263)	2,784 (221)	2,739 (275)	2,769 (261)
Carbohydrate (kcal)	1,934 (781)	2,082 (711)	2,041 (633)	1,888 (662)	1,990 (702.23)
Protein (kcal)	472 (199)	505 (182)	541 (200)	457 (159)	505 (182)
Fat (kcal)	1,457 (679)	1,555 (632)	1,550 (593)	1,345 (528)	1,483 (617)

Values are expressed as means ± standard deviations or *n* (%) unless specified otherwise. Dietary intake variables are reported in kcals/day unless otherwise specified. Significant differences between seasons were tested using an ANOVA, and global *p*-values are denoted, overall *p < 0.05. AA, African American; AI/AN, American Indian and Alaska Native; FFM, fat-free mass; FM, fat mass; WMEN, weight-maintaining energy needs.

### 3.1 Determinants of dietary intake

FFM index was a significant positive predictor of all energy intake measures: total (*β* = 173 kcal, *p* < 0.0001), %WMEN (*β* = 5%, *p* = 0.002), protein (*β* = 28 kcal, *p* < 0.0001), fat (*β* = 83 kcal, *p* < 0.0001), and carbohydrate (*β* = 69 kcal, *p* = 0.0017). In addition, SPA was also a significant positive predictor in all energy intake models: total (*β* = 37 kcal, *p* = 0.0033), %WMEN (*β* = 1%, *p* = 0.011), protein (*β* = 5 kcal, *p* = 0.0042), fat (*β* = 13 kcal, *p* = 0.020), and carbohydrate (*β* = 20 kcal, *p* = 0.0033). Conversely, FM index was consistently a significant negative predictor of intake: total (*β* = −101 kcal, *p* = 0.0016), %WMEN (*β* = −5%, *p* < 0.0001), protein (*β* = −17 kcal, *p* = 0.0002), fat (*β* = −44 kcal, *p* = 0.0024), and carbohydrate (*β* = −44 kcal, *p* = 0.0095). These determinants mostly remained significant in adjusted models assessing secular and seasonal trends (Tables 3, 4).

**TABLE 3:** General linear models adjusted secular trends demonstrating the significance of determinants of dietary intake.

Model predictors	Total intake (kcal) *β* (*p*-value)	Total intake (% WMEN^a^) *β* (*p*-value)	Protein (kcal) *β* (*p*-value)	Fat (kcal) *β* (*p*-value)	CHO (kcal) *β* (*p*-value)
FFM index	141.9 (0.0002)	3.5 (0.012)	19.8 (< 0.0001)	59.7 (0.0006)	62.4 (0.0023)
FM index	−55.1 (0.065)	−3.06 (0.0043)	−7.7 (0.049)	−15.3 (0.26)	−31.04 (0.0003)
SPA	35 (0.0036)	1.09 (0.012)	4.9 (0.0032)	12.7 (0.021)	18.7 (0.0043)

This table demonstrates information for models adjusted in two different ways, differentiated by the horizontal line. The FFM Index and FM Index models are adjusted for secular trends, age, sex, race, FFM index, and FM index, while the SPA model includes those same adjustments plus SPA (*n* = 206). The *β* and *p*-values for the GLMs are reported. *β* are reported as the change in kcal/ 1 unit change in the predictor, except for %WMEN, which is expressed as % of calculated WMEN/ 1 unit change in the predictor. *^a^*weight-maintaining energy needs.

**TABLE 4:** General linear models adjusted seasonality demonstrating the significance of determinants of dietary intake.

Model predictors	Total intake (kcal) *β* (*p*-value)	Total intake (% WMEN^a^) *β* (*p*-value)	Protein (kcal) *β* (*p*-value)	Fat (kcal) *β* (*p*-value)	CHO (kcal) *β* (*p*-value)
FFM index	219.05 (< 0.0001)	6.40 (< 0.0001)	34.08 (< 0.0001)	98.84 (< 0.0001)	93.75 (< 0.0001)
FM index	−108.63 (0.0002)	−5.11 (< 0.0001)	−17.63 (< 0.0001)	−42.32 (0.0017)	−52.71 (0.0007)
SPA	32 (0.010)	1.00 (0.028)	4.43 (0.013)	11.32 (0.048)	17.38 (0.0097)

This table demonstrates information for models adjusted in two different ways, differentiated by the horizontal line. The FFM Index and FM Index models are adjusted for season, age, sex, race, FFM index, and FM index, while the SPA model includes those same adjustments plus SPA (*n* = 206). The *β* and *p*-values for the GLMs are reported. *β* are reported as the change in kcal/ 1 unit change in the predictor, except for %WMEN, which is expressed as % of calculated WMEN/ 1 unit change in the predictor. *^a^*weight-maintaining energy needs.

### 3.2 Seasonality trends of dietary intake and related body composition measures

Overall, there was no significant effect of season in any measure of intake, in either unadjusted (Table 5) or adjusted models. In adjusted models, there were no seasonal differences in total kcal intake (overall *p*-value for season = 0.10), %WMEN (overall *p*-value for season = 0.061), fat intake (overall *p*-value for season = 0.12), carbohydrate intake (overall *p*-value for season, *p* = 0.16), and protein intake (overall *p*-value for season, *p* = 0.024, all post-hoc *p* > 0.05).

**TABLE 5:** Unadjusted general linear models demonstrating a lack of significance between measures of intake and seasons.

Model predictors	Total intake (kcal) *β* (*p*-value)	Total intake (% WMEN^a^) *β* (*p*-value)	Protein (kcal) *β* (*p*-value)	Fat (kcal) *β* (*p*-value)	CHO (kcal) *β* (*p*-value)
Fall	−249.32 (0.26)	−11.11 (0.13)	−33.49 (0.27)	−97.83 (0.32)	−147.78 (0.19)
Spring	2.34 (0.99)	−0.45 (0.95)	36.08 (0.24)	−4.61 (0.96)	−41.60 (0.72)
Summer	−415.49 (0.079)	−14.15 (0.071)	−48.29 (0.13)	−209.28 (0.048)	−194.42 (0.11)

This table demonstrates information for unadjusted models. The *β* and *p*-values for the GLMs are reported. *β* are reported as the change in kcal/ season when compared to the reference season of Winter, except for %WMEN, which is expressed as % of calculated WMEN/ season. *^a^*weight-maintaining energy needs.

### 3.3 Secular trends of body composition

The secular trend in body composition metrics was analyzed to assess whether there were any concurrent changes in body composition during the analyzed changes in dietary intake. Separate models were adjusted for sex, age, race/ethnicity, and date, with body size or composition as the dependent variable. Over time, there was no significant change in weight (*β* = 0.099 kg/year, *p* = 0.70), BMI (*β* = −0.10 kg/m^2^/year, *p* = 0.15), and body fat (%) (*β* = −0.023%/year, *p* = 0.37).

### 3.4 Secular trends of dietary intake

**FIGURE 2:**
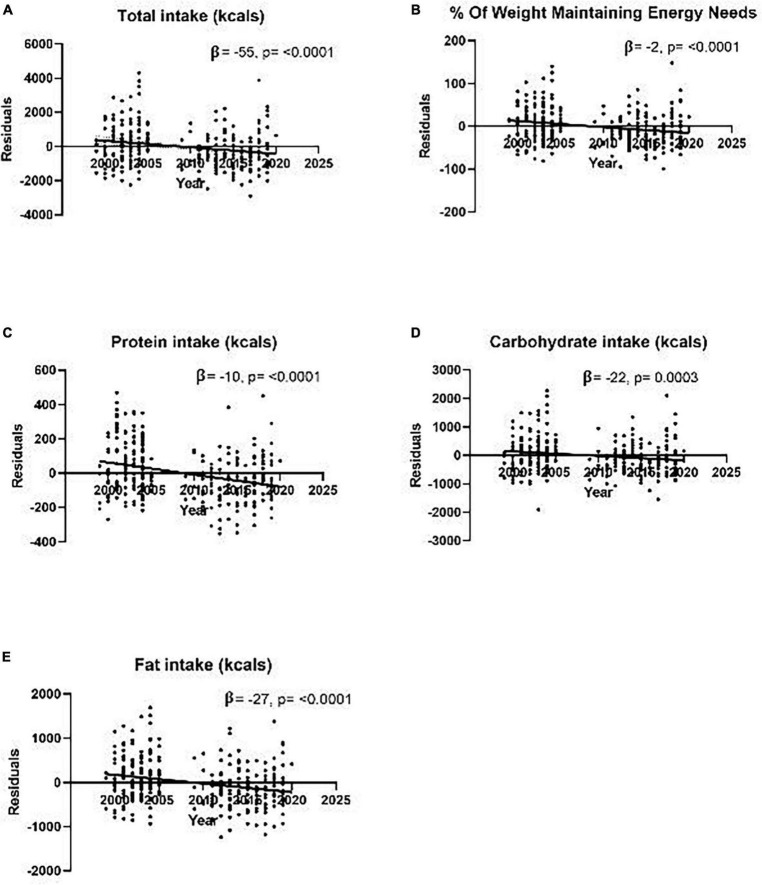
Graphs of adjusted residual models for secular trends analyses of various measures of dietary intake as measured by an objective vending machine paradigm from 1999 to 2020 during a 10-day inpatient study. Models are adjusted for age, sex, race, FFM index, FM index, and date and demonstrate the secular trend in **(A)** residual total calorie intake (*r*^2^ = 0.36) **(B)** residual %WMEN (*r*^2^ = 0.25) **(C)** residual total protein intake in kcals (*r*^2^ = 0.42) **(D)** residual total carbohydrate intake reported in kcals (*r*^2^ = 0.30) **(E)** residual total fat intake reported in kcals (*r*^2^ = 0.35). *β* are reported as the change in kcals/year. No participants were recruited for this study from 2006 to 2008, leaving a small break in the middle, as seen in the graphs above.

Unadjusted models for total energy intake, %WMEN, fat, carbohydrate, and protein intake were negatively associated with time (Table 6). The effect size in these GLMs for intake variables was expressed as change in kcals over a year. After adjustments for age, race/ethnicity, sex, FFM index, and FM index, secular decreases in total energy intake (*β* = −55 kcal/year, *p* < 0.0001), %WMEN (*β* = −2%/year, *p* < 0.0001), protein intake (*β* = −10 kcal/year, *p* < 0.0001), fat intake (*β* = −27 kcal/year, *p* < 0.0001), and carbohydrate intake (*β* = −22 kcal/year, *p* = 0.0003) remained significant (Figure 2). Additional models were run with percent total energy intake by macronutrient instead of total macronutrient calories, and results remained largely similar (Table 7).

**TABLE 6:** General linear models with varying adjustments demonstrating a decline in *ad libitum* intake and the significance of the adjustments in the models from 1990 to 2020.

Intake measure	(A) Unadjusted Model *β* (R^2^)	(A) *r*	(B) Adjusted Model *β* (R^2^)	(B) partial *r*	(C) Adjusted plus SPA Model *β* (R^2^)	(C) partial *r*
Total intake (kcal)	−80** (0.16)	−0.40**	−55** (0.36)	−0.27**	−51** (0.37)	−0.28**
Total intake (% WMEN^a^)	−3** (0.15)	−0.39**	−2** (0.25)	−0.28**	−1** (0.28)	−0.29**
Protein (kcal)	−13** (0.21)	−0.45**	−10** (0.42)	−0.37**	−10** (0.40)	−0.38**
Fat (kcal)	−40** (0.18)	−0.42**	−27** (0.35)	−0.30**	−23** (0.35)	−0.29**
CHO (kcal)	−35** (0.12)	−0.34**	−22* (0.30)	−0.21*	−23** (0.32)	−0.24*

This table demonstrates information for models adjusted in three different ways. The *β* and *R*^2^ for the GLMs are reported. Correlation coefficients (*r*) or partial *r* are also reported to demonstrate the impact of further adjustments to the relationship between intake and time. Model (A) is an unadjusted GLM for secular trends and dietary intake. While (B) is adjusted for age, sex, race, FFM index, and FM index, then (C) is further adjusted for SPA, *n* = 206. *β* are reported as the change in kcals/year, except for %WMEN, which is expressed as %/year. **p < 0.0001, *p < 0.05. *^a^*Weight maintaining energy needs.

**TABLE 7:** General linear models with varying adjustments demonstrating a decline in *ad libitum* percent macronutrient intake and variance in the models from 1990 to 2020.

Intake measure	(A) Unadjusted Model *β*	(A) R^2^	(B) Adjusted Model *β*	(B) R^2^	(C) Adjusted plus SPA Model *β*	(C) R^2^
Protein (%)	−0.00066**	0.023	−0.00077*	0.16	−0.00080*	0.15
Fat (%)	−0.022**	0.051	−0.0020*	0.10	−0.00099	0.056
CHO (%)	0.0015**	0.026	0.00062*	0.090	0.00069	0.041

This table demonstrates information for models adjusted 3 different ways. The *β* and *R*^2^ for the GLMs are reported. Model (A) is an unadjusted GLM for secular trends and dietary intake. While (B) is adjusted for age, sex, race, FFM index, and FM index, then (C) is further adjusted for SPA, *n* = 206. *β* are reported as the change in % energy intake from each macronutrient/year **p < 0.0001, *p < 0.05.

### 3.5 Secular trends of dietary intake and SPA

In previous studies, SPA demonstrated a significant change over time and was therefore added to time and intake models (15). After including SPA, time remained significant in all intake models: total (*β* = −51 kcal/year, *p* < 0.0001), %WMEN (*β* = −2%/year, *p* < 0.0001), protein (*β* = −10 kcal/year, *p* < 0.0001), fat (*β* = −23 kcal/year, *p* < 0.0001), and carbohydrate (*β* = −23 kcal/year, *p* < 0.0001). Interestingly, as seen in Table 2, including SPA in the models led to a decline in effect size while remaining a significant positive predictor or intake.

## 4 Discussion

In this analysis of food intake data over twenty years, while season had no effect, there was an overall secular decline in *ad libitum* food intake in all recorded measures. FFM index and SPA were positive determinants of energy intake and accounted for a large proportion of the variance. With each further adjustment of the secular trends models to include these determinants of intake, there was a decline in overall effect size with a concomitant increase in R^2^. Despite SPA and body composition adjustment, the secular decrease in intake was still present. In addition, there were no changes in body composition measurements, including body weight, BMI, and body fat percentage.

Previous studies in our unit have associated both SPA and FFM index with dietary intake (5, 16). In this case, SPA reflects small restless physical movements participants make in the respiratory chambers, like sitting to standing, and is a tiny yet highly variable component of energy expenditure (15). In all season and secular models, FFM index and SPA were significant positive predictors of intake and thus were essential features of each model. Models were also adjusted for FFM, FM, and height separately, as opposed to FFM index, and significance remained (data not shown). Other secular and seasonal analyses mentioned above, such as Brown et al., used NHANES cross-sectional data and did not adjust for these known determinants in their models (1, 13, 29, 30). As shown in Table 6, the secular decline in intake persisted despite adjustment for these parameters. With each model adjustment, the parameter estimates decreased, but overall R^2^ increased, indicating that the FFM index and SPA accounted for some of the decline. While the FFM index was a significant positive predictor in dietary intake models, the FM index was a significant negative predictor of intake. While this is unexpected, other research from our unit found similar results and attributed them to the potential interactions with hormones in obese individuals (16).

Previous studies have reported mixed results on the seasonality of dietary intake (6–10). Winter has been significantly associated with increased dietary intake in several studies (6–8). It is worth noting that while Phoenix, the location of the above analysis, can be characterized by a milder winter compared to these other studies, results in winter have been inconsistent nonetheless. In these studies, seasonal effects varied, with individuals in the US eating more in winter and Japanese eating less (6). Several other analyses have found no significant relationships between dietary intake and season (9, 10). Studies demonstrating seasonal changes in dietary intake relied on methods such as 24-h recall, dietary questionnaires, and food diaries (6–10). In contrast, this above analysis used objective measures of dietary intake on a validated vending machine paradigm (17). There was no overall effect of season for overall or macronutrient intake, even in models adjusted for confounders such as FFM-index or SPA.

When considering these known determinants, the secular trends analysis results showed an unexpected decline in intake across all macronutrient intake measures. Previous reports demonstrating temporal trends in dietary intake are varied. Shan et al. (13) also reported decreases in carbohydrate kcal intake over a similar period, from 1999 to 2016. However, they found increases in protein and fat kcals. (13) In contrast, Brown et al. (1) found increases in carbohydrate intake and decreases in protein and fat. Lastly, Ford and Dietz (29) reported an increase in overall kcals from the 1971–1975 NHANES to the 2003–2004 NHANES but a significant decrease from the 2009–2010 NHANES. Ford and Dietz (29) concluded that dietary intake appears to peak in the 2003–2004 NHANES data and is beginning to trend downward (29). A similar trend was demonstrated with added sugar by Wang et al. (30), with added sugar intake peaking in 2000–2002 and declining by 2009–2010.

A potential explanation for this decrease in measures of intake lies in previously published data on the decrease in SPA. In several previously reported studies, as well as our results above, intake was significantly positively associated with SPA that is limited to restless and fidgeting-like behaviors possible in the whole-room calorimeter (1, 5). Additionally, Travis et al. (15) demonstrated a secular decline in SPA. If the decline in SPA is representative of an overall decrease in physical activity, this could be reflected in less drive to eat (e.g., reduced activity reducing drive for intake). Thus, the secular trend described may be an adaptation to unhealthy and increasingly sedentary conditions. This trend toward increasing sedentary behavior, which, despite reduced energy intake, may be why there aren’t accompanying body composition changes. However, Travis et al. (15) also found significant secular decreases in BMI to accompany their decreases in SPA. Interestingly, no changes in body composition measures (BMI, weight, and percent body fat) were found in this current analysis.

Another possible explanation for the secular decline in energy intake may be increased awareness of nutrition science and government health promotion during this time (11, 31). In particular, the emphasis on overconsumption as a public health issue (32). The beginning of the 21st century was a time marked by an obesogenic food environment of unlimited access to highly palatable foods (12). While we still live in this environment today, the public discourse around the health implications of this environment has increased. This can potentially impact a participant’s eating behavior in controlled conditions. In fact, one study demonstrated that participants tended to eat more at home than in the lab (33). Previous research has also shown that participants who are being watched or know they are being watched tend to restrict their calories (33). Therefore, participants under experimental conditions may have an unintentional bias and increased intake awareness, leading to an inadvertent restriction in energy intake under controlled conditions. This may offer an additional or alternate reason for participants’ eating less on our vending machines over time, without a corresponding decrease in weight or BMI. Unfortunately, this is something that could not be measured in this present analysis but is an interesting consideration for researchers moving forward.

There are a few limitations of this study that need to be acknowledged. First, the population was predominantly Indigenous American, thus possibly limiting the generalizability of these results. However, in other studies from our unit, similarities in the physiology of energy intake have been demonstrated across populations (34). Secondly, food consumption in this vending machine paradigm is done in an inpatient setting, thus not in line with standard dietary intake in a free-living population. However, the vending machine paradigm has high reproducibility across repeated visits (ICC = 0.90), indicating people consistently eat the same across vending days (17). Additional information on secular or seasonal changes in satiety may have provided further insight into dietary intake trends but unfortunately was not collected in this study. Lastly, similar to our food intake measurement, spontaneous physical activity was measured under controlled conditions in the respiratory chamber. Therefore, this may not be entirely reflective of an individual’s free-living physical activity. However, since the measurement of spontaneous physical activity (SPA) has not changed significantly in this time, it may still demonstrate an overall trend of less physical activity while in the chamber, as previously published (5).

Herein, we report a secular decrease in *ad libitum* intake during an inpatient research study. Increased public health awareness around excessive dietary intake, in addition to previously published work showing a decline in SPA, may be factors driving this current observation of a decrease in energy intake. This suggests that when conducting dietary intake measurements in controlled settings, one should consider the possibility of unintentional dietary restriction among participants. Overall, these findings may elucidate trends in energy intake, its determinants over time, and can inform future analyses into this relationship.

## Data availability statement

The datasets presented in this article are not readily available due to the enrollment of Indigenous Americans of Southwestern heritage. Requests to access the datasets should be directed to SV, votrubas@niddk.nih.gov.

## Ethics statement

The studies involving humans were approved by the National Institute of Health IRB. The studies were conducted in accordance with the local legislation and institutional requirements. The participants provided their written informed consent to participate in this study.

## Author contributions

MA: Formal analysis, Visualization, Writing – original draft, Writing – review & editing. ES: Data curation, Formal analysis, Visualization, Writing – original draft, Writing – review & editing. PP: Formal analysis, Writing – original draft, Writing – review & editing. JK: Conceptualization, Data curation, Formal analysis, Funding acquisition, Investigation, Methodology, Project administration, Supervision, Writing – original draft, Writing – review & editing. SV: Conceptualization, Formal analysis, Funding acquisition, Investigation, Methodology, Project administration, Resources, Supervision, Writing – original draft, Writing – review & editing.
